# Extracellular Matrix Expression in Human Pancreatic Fat Cells of Patients with Normal Glucose Regulation, Prediabetes and Type 2 Diabetes

**DOI:** 10.3390/ijms241311169

**Published:** 2023-07-06

**Authors:** Dorothea Siegel-Axel, Morgana Barroso Oquendo, Felicia Gerst, Falko Fend, Robert Wagner, Martin Heni, Alfred Königsrainer, Hans-Ulrich Häring, Andreas Fritsche, Erwin Schleicher, Andreas L. Birkenfeld, Norbert Stefan

**Affiliations:** 1 Institute for Diabetes Research and Metabolic Diseases (IDM) of the Helmholtz Center Munich at the University of Tübingen, 72076 Tübingen, Germanynorbert.stefan@med.uni-tuebingen.de (N.S.); 2 German Center for Diabetes Research (DZD e.V.), 85764 Neuherberg, Germany; 3 Department of Internal Medicine IV, University Hospital of Tübingen, Otfried-Müller Str. 10, 72076 Tübingen, Germany; 4 EKU Tübingen, Quantitative Biology Center (QBiC), University of Tübingen, 72076 Tübingen, Germany; 5 Department of General Pathology and Pathological Anatomy, University Hospital Tübingen, 72076 Tübingen, Germany; 6 Institute for Clinical Diabetology, German Diabetes Center (DDZ), Heinrich Heine University Düsseldorf (HHU), 40225 Düsseldorf, Germany; 7 Division of Endocrinology and Diabetology, Department of Internal Medicine I, University Hospital Ulm, 89081 Ulm, Germany; 8 Department of General, Visceral and Transplant Surgery, University Hospital Tübingen, 72076 Tübingen, Germany; 9 Institute for Clinical Chemistry and Pathobiochemistry, Department for Diagnostic Laboratory Medicine, University Hospital Tübingen, 72076 Tübingen, Germany

**Keywords:** (pre)adipocytes, islets, fetuin-A, monocytes, extracellular matrix, adipose tissue, diabetes

## Abstract

Previously, we found that human pancreatic preadipocytes (PPAs) and islets influence each other and that the crosstalk with the fatty liver via the hepatokine fetuin-A/palmitate induces inflammatory responses. Here, we examined whether the mRNA-expression of pancreatic extracellular matrix (ECM)-forming and -degrading components differ in PPAs from individuals with normal glucose regulation (PPAs-NGR), prediabetes (PPAs-PD), and type 2 diabetes (PPAs-T2D), and whether fetuin-A/palmitate impacts ECM-formation/degradation and associated monocyte invasion. Human pancreatic resections were analyzed (immuno)histologically. PPAs were studied for mRNA expression by real-time PCR and protein secretion by Luminex analysis. Furthermore, co-cultures with human islets and monocyte migration assays in Transwell plates were conducted. We found that in comparison with NGR-PPAs, TIMP-2 mRNA levels were lower in PPAs-PD, and TGF-β1 mRNA levels were higher in PPAs-T2D. Fetuin-A/palmitate reduced fibronectin, decorin, TIMP-1/-2 and TGF-ß1 mRNA levels. Only fibronectin was strongly downregulated by fetuin-A/palmitate independently of the glycemic status. Co-culturing of PPAs with islets increased TIMP-1 mRNA expression in islets. Fetuin-A/palmitate increased MMP-1, usherin and dermatopontin mRNA-levels in co-cultured islets. A transmigration assay showed increased monocyte migration towards PPAs, which was enhanced by fetuin-A/palmitate. This was more pronounced in PPAs-T2D. The expression of distinct ECM components differs in PPAs-PD and PPAs-T2D compared to PPAs-NGR, suggesting that ECM alterations can occur even in mild hyperglycemia. Fetuin-A/palmitate impacts on ECM formation/degradation in PPAs and co-cultured islets. Fetuin-A/palmitate also enhances monocyte migration, a process which might impact on matrix turnover.

## 1. Introduction

In the past 10 years, several studies revealed important knowledge about the role of different fat depots for glucose metabolism [1]. In this respect, we assessed the expression and secretion pattern of cytokines, chemoattractants, growth factors, and adipokines in subcutaneous, visceral, perivascular, renal sinus, and pancreatic preadipocytes (PPAs) and differentiated pancreatic adipocytes (PAs) and investigated their cross-talk with adjacent cells. Furthermore, we studied this interaction based on the presence and absence of fatty liver-derived hepatokine fetuin-A [2,3,4,5,6]. We focused on fetuin-A because this protein is upregulated in inflamed fatty liver and fetuin-A bound to fatty acids induces subclinical inflammation and insulin resistance [7,8,9,10]. In this research field, it is important to acknowledge that beside cells, mature tissues also contain a great variety of extracellular matrix (ECM) proteins [11]. The ECM is built by variable structures which determine the tissue architecture and form a mechanical scaffold for adhesion, migration, and cellular function. Consequently, fat cell differentiation and function are influenced by ECM and growth factors [12], and the upregulation of ECM components is closely related to adipogenesis [13]. Interstitial fibers and pericellular basement membranes are predominantly built by collagens [14]. Interstitial fibers and pericellular basement membranes are predominantly built by collagens and elastin, forming rigid and elastic bundles which provide the major ECM scaffold to sustain the structure, maintenance, differentiation and function of adipose tissue [15,16]. About 20 chemically distinct collagen (Col) forms have been described [17]. Furthermore, the cross-talk of fat cells with ECM can activate intracellular signaling pathways in both PPAs and differentiated PAs [12], and the signaling of ECM proteins and growth factors influences cellular survival, proliferation, and differentiation [18].

The ECM also plays an important role in islet function and survival [19]. Col IV and VI are major components of the peri- and intra-islet basement membrane [20]. To our knowledge, it has not been established whether the expression of different ECM components in pancreatic adipose tissue has an impact on islet function. In the present study, we isolated PPAs from patients with normal glucose regulation (PPAs-NGR), prediabetes (PPAs-PD), and type 2 diabetes (PPAs-T2D) and examined the expression level of a large panel of ECM-forming and -degrading components, as well as the effects of fetuin-A and palmitate, on pancreatic fat cell ECM expression and immune cell invasion in vitro. Moreover, in order to assess whether fatty liver–fatty pancreas cross-talk may alter the composition of islet ECM, we evaluated the impact of isolated PPAs on the ECM of co-cultured human islets in the presence of fetuin-A and palmitate. 

## 2. Results

### 2.1. Detection of ECM and Monocytes/Macrophages in Pancreatic Resections

**Figure 1:**
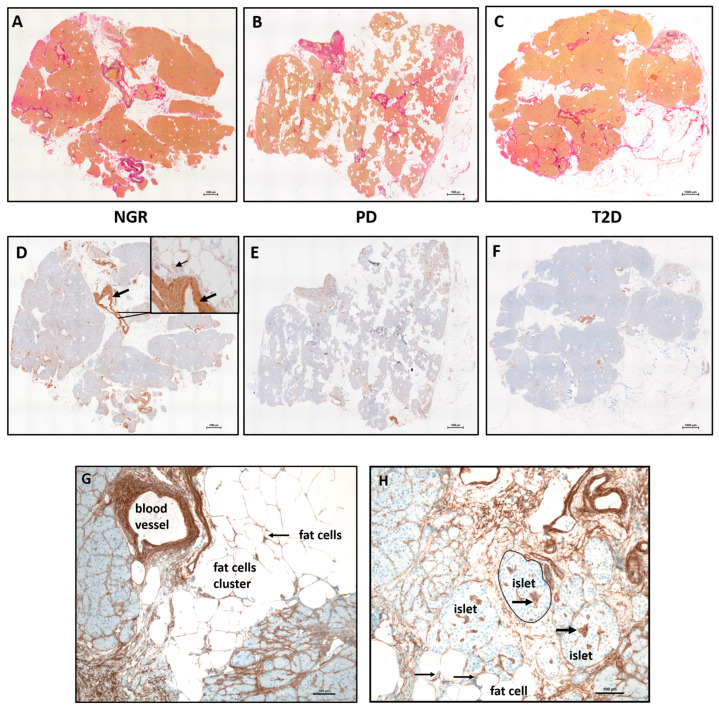
Histology of human pancreatic tissue sequential sections of subjects with (**A**,**D**) NGR, (**B**,**E**) PD, and (**C**,**F**) T2D. The stainings were performed at serial sections. (**A**–**C**) Elastica–van Gieson staining of Col I, III, and elastin. Accumulation or invasion of fat cells (white) is visible in all samples. (**D**–**F**) Immunostaining against the basement membrane protein Col IV. The specific brown staining is predominantly visible in the vessel wall of the arteries (black arrow, (**D**)). (**G**) The blood vessels are often located near fat cell clusters and contain a large amount of Col IV in their wall. (**H**) Col IV is present around each fat cell (thin black arrows) and inside islets (thick black arrows). The scale bars on the lower right side represent 1000 µm and 100 µm.

Elastica van-Gieson stainings were performed in order to detect connective tissue (predominantly collagens) and elastic fibers (Figure 1A–C, red). Using immunocytochemical staining against Col IV, the basal lamina (brown) could be detected in samples of all subgroups (Figure 1D–F) but was predominately in blood vessels (thick arrow, Figure 1D insert) and fat cell clusters (thin arrow, Figure 1D insert). Accumulation of Col IV is more obvious around blood vessels, near fat cell clusters, and around the fat cells (Figure 1G). Col IV was also detected around and inside islets (Figure 1H). In general, samples from all subjects were very heterogeneous in terms of ECM content.

### 2.2. RNA Expression of ECM Components in PPAs

**Figure 2:**
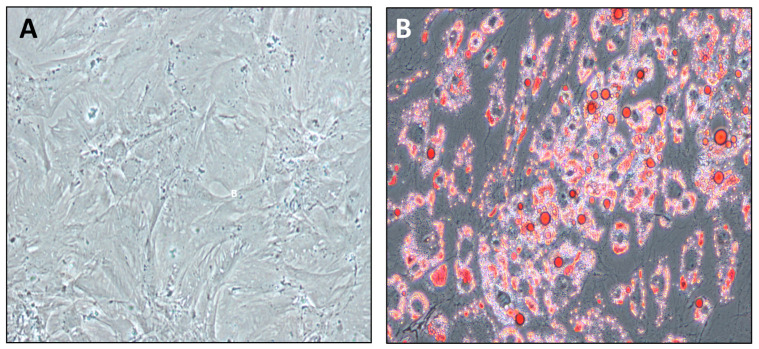
(**A**) Characteristic growth pattern of a representative PPA culture. (**B**) Oil-red O staining intercellular triglycerides in differentiated, mature PAs. About 95% of cells were fully differentiated into PAs.

PPAs from pancreatic resections of patients with NGR, PD, or T2D were isolated and subcultured. These primary cells showed the characteristic spindle shape, with cytoplasmic protrusions and cluster formation (Figure 2A). PPAs were used for all experiments in the second passage, aiming to maintain most of their in vivo characteristics. Following exposure to differentiation medium, the cytoplasm of fat cells was packed with lipids, the characteristic of mature, differentiated adipocytes (Figure 2B). 

**Figure 3:**
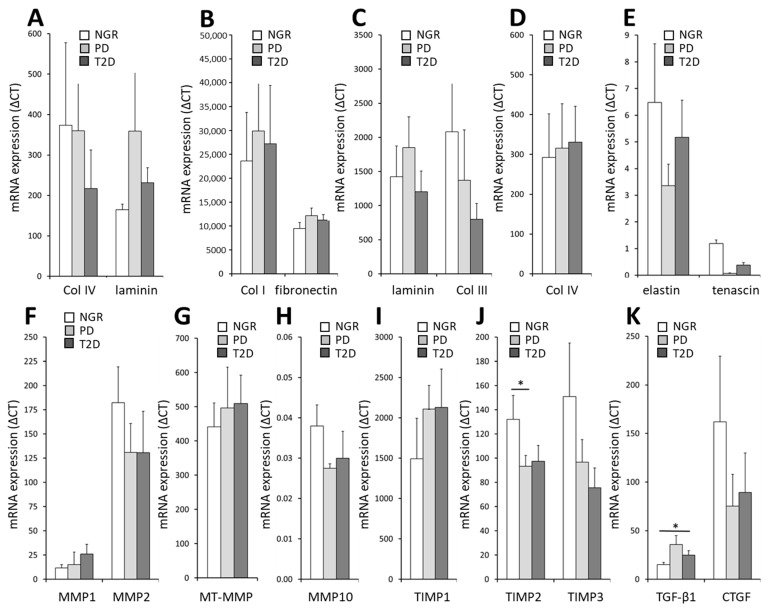
(**A**–**K**) Relative mRNA levels of a variety of ECM proteins, matrix-degrading proteins, and associated growth factors in PPAs from patients with NGR (white bars), PD (grey bars), and T2D (black bars). Results are expressed as the mean ± SEM (*n* = 5–7, * *p* < 0.05).

RT-PCR analysis revealed that the mRNA level of the ECM components Col I, III, IV, and VI, decorin, laminin, elastin, and tenascin were similar in PPAs isolated from patients with NGR, PD, or T2D (Figure 3A–E). As the amount and composition of ECM is regulated by matrix metalloproteinases and their inhibitors, we also measured the mRNA level of such proteins. The TIMP-2 mRNA was lower in PPAs-PD compared to PPAs-NGR (Figure 3J). The mRNA levels of other MMPs or TIMPs were similar in the PPAs-NGR, PPAs-PD, and PPAs-T2D groups, respectively (Figure 3F–J). In addition, we measured the mRNA levels of TGF-β1 and CTGF (Figure 3K), two growth factors important for matrix remodeling. While the mRNA of CTGF was similar in the PPAs from the three groups, the TGF-β1 mRNA level was higher in PPAs-PD compared to PPAs-NGR and tended to be higher in PPAs-T2D compared to PPAs-NGR (Figure 3K). Taken together, these findings indicate that the pancreatic fat cells appear to be conditioned by the metabolic milieu of the donor for the expression of distinct ECM components. 

### 2.3. Differences in ECM Expression between PPAs and PAs: Changes of mRNA Expression during Differentiation from PPAs to PAs

**Figure 4:**
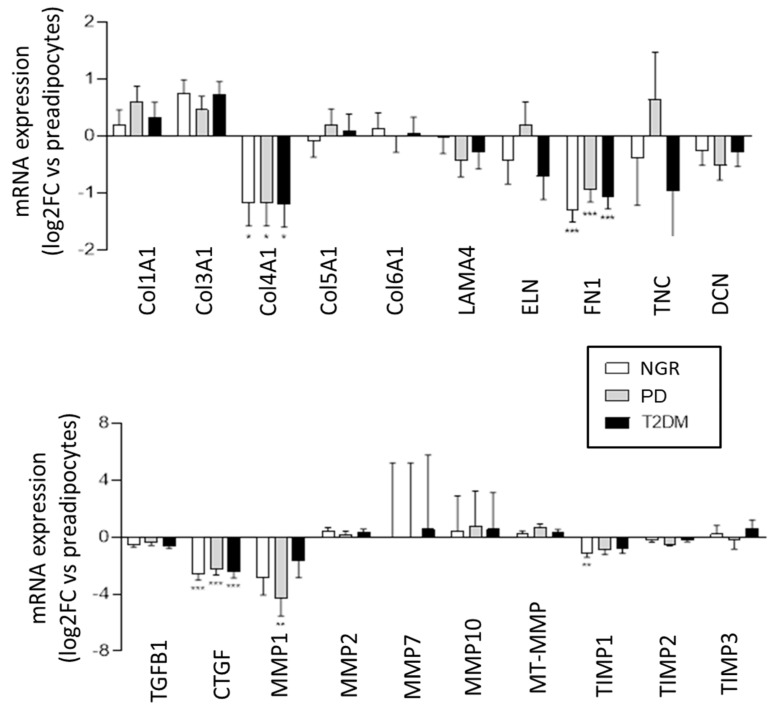
Differential expression (Log2–fold change; PAs versus PPAs) of ECM-associated proteins and their regulators driven by pancreatic adipocyte differentiation obtained from RNA-seq data. PPAs-NGR (*n* = 4, white bars), PPAs-PD (*n* = 4, grey bars), or PPAs-T2D (*n* = 4, black bars) were differentiated into PAs. * *p* < 0.05, ** *p* < 0.01, *** *p* < 0.001 vs. PPAs.

To evaluate if the PPAs are conditioned by the in situ metabolic milieu, or if inherited factors govern the expression of ECM components and its regulators, a comparative expression profiling of PPAs and differentiated PAs from individuals with NGR, PD, and T2D was conducted via RNAseq (Figure 4). During the differentiation of PPAs to mature PAs, a significant and nearly identical decrease in mRNA expression was found for Col IVA1, fibronectin (FN1), and CTGF, in all three groups, and for MMP1, which was statistically significant only in the PD group. All other ECM components and their regulators were not significantly different. These results suggest that the alterations of the expression pattern of ECM components during the differentiation of PPAs to PAs are independent of the donor’s metabolic environment or that the differences in ECM expression observed in the PPAs of the three patient groups are lost during differentiation. 

### 2.4. Effects of Fetuin-A/Palmitate on mRNA Expression and Protein Secretion of ECM Components in PPAs

**Figure 5:**
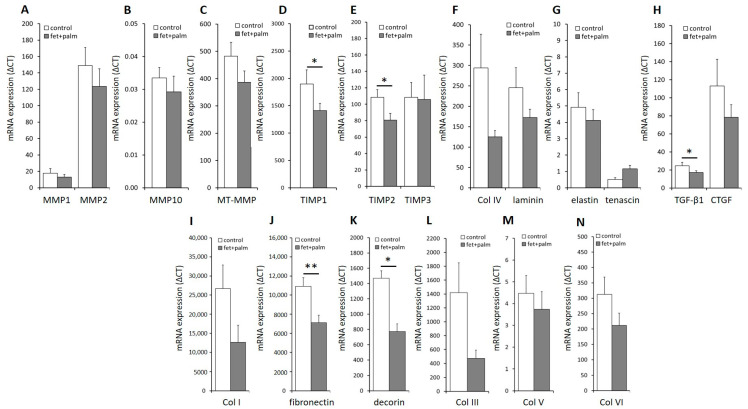
(**A**–**N**) Relative mRNA levels in PPAs after treatment with 600 μg/mL fetuin-A + 60 µmol/L palmitate for 24h (black bars) versus untreated controls (white bars). Data from all of the individuals of the 3 groups are pooled. ΔCT values are shown (related to the housekeeping gene *RPS13*. Data are expressed as the mean ± SEM (*n* = 5–7, Student’s *t*-test, * *p* < 0.05, ** *p* < 0.01).

In order to evaluate the impact of the prediabetic milieu on PAs, we challenged the PPAs with a combination of palmitate and fetuin-A. In PPAs treated for 24 h with palmitate and fetuin-A, a moderate downregulation of the mRNA expression of some matrix proteins was observed (Figure S1). Interestingly, a significant downregulation of the mRNA expression of TIMP1 and TIMP2 (Figure 5D,E), TGF-ß1 (Figure 5H), fibronectin (Figure 5J), and decorin (Figure 5K) was found when cells from all individuals were combined for analysis. The strong downregulation of the mRNA of fibronectin was observed in each group of PPAs isolated from subjects with NGR, PD, or T2D (Supplementary Figure S1 and Table 1). Our results indicate that a prediabetic milieu, e.g., consisting of elevated fetuin-A/palmitate, reduces the expression of several ECM components and their modulating or regulating proteins. 

**Figure 6:**
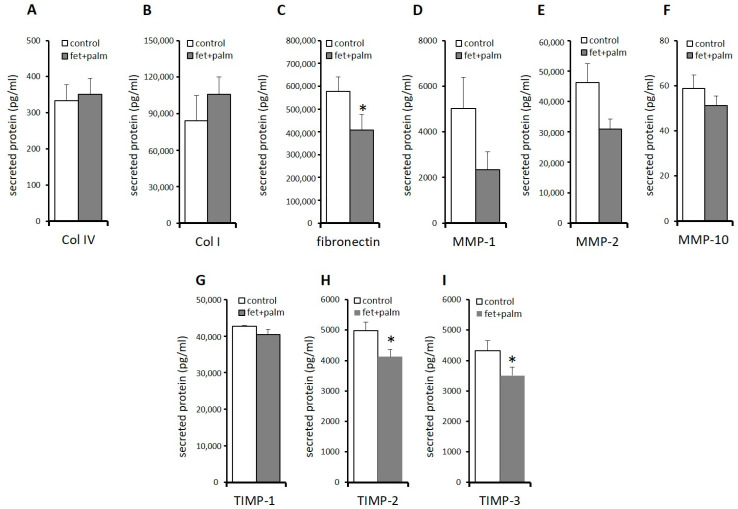
(**A**–**I**) Protein levels in PPAs after treatment with 600 μg/mL fetuin-A + 60 μmol/L palmitate for 24 h (dark grey bars) versus untreated controls (white bars). The data from all individuals in the 3 groups are pooled. Protein concentrations were (pg/mL) determined using the Luminex technology (see methods). Data are expressed as the mean ± SEM (*n* = 5–7, Student’s *t*-test, * *p* < 0.05).

In order to support the results found on mRNA levels, we determined the protein concentrations in the supernatant of various ECM components and ECM modulating factor (Figure 6). A significant decrease in fibronectin (Figure 6C) and TIMP-2 (Figure 6H) levels was found in the supernatants of PPAs after treatment with fetuin-A/palmitate compared to untreated controls, which reflects the significantly decreased fibronectin and TIMP-2 mRNA expression (Figure 5). TIMP-1 protein levels decreased also after fetuin-A/palmitate treatment but without reaching significancy (Figure 6G), which might be explained by the low sample size. Decorin protein levels could not be determined because decorin antibody beads were not included in the Human Magnetic Luminex Assay (R&D systems 9-Plex array). An additional Western blot or single ELISA analysis was not possible because of a lack of sufficient cell culture supernatant.

### 2.5. Effects of Fetuin-A/Palmitate on mRNA Expression of ECM Proteins in Co-Cultured Islets

**Figure 7:**
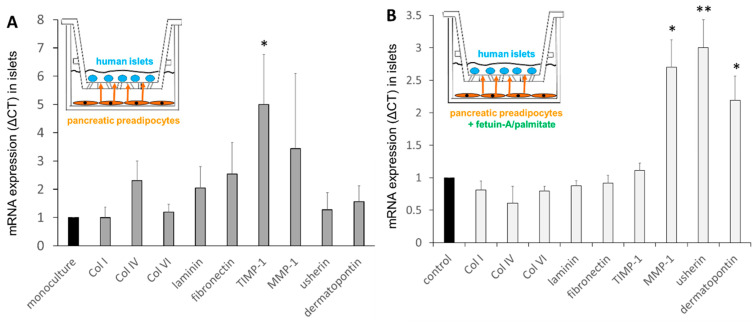
Relative mRNA levels of human islets in mono-cultures and in co-cultures with PPAs, as well as under the influence of 600 μg/mL fetuin-A/60 μmol/L palmitate for 24 h. (**A**) The effect of co-cultured PPAs on islet mRNA levels (fold-change, grey bars) compared with respective mRNA levels in mono-cultured islets (black bar, defined as 1, under the same conditions). (**B**) The effect of co-cultured PPAs and fetuin-A/palmitate treatment on islet mRNA levels (fold-change) compared with untreated controls (black bars, defined as 1) under the same conditions. Data are expressed as the mean ± SEM (*n* = 3, Student’s *t*-test, * *p* < 0.05, ** *p* < 0.01).

In order to examine the paracrine cross-talk between human PPAs and islets, an in vitro co-culture system was established, as described previously [2,3]. The presence of PPAs with islets in co-cultures resulted in a significant increase in TIMP-1 mRNA levels in islets (Figure 7A). In order to investigate whether the effects of PPAs on islets are influenced by the fatty liver-derived hepatokine fetuin-A and palmitate, co-cultures were exposed to fetuin-A/palmitate. The mRNA levels of Col I, IV, VI, and laminin were not altered in co-cultured islets, whereas those of MMP-1, usherin, and dermatopontin were upregulated (Figure 7B). 

### 2.6. Detection of Monocytes/Macrophages in Pancreatic Resections

**Figure 8:**
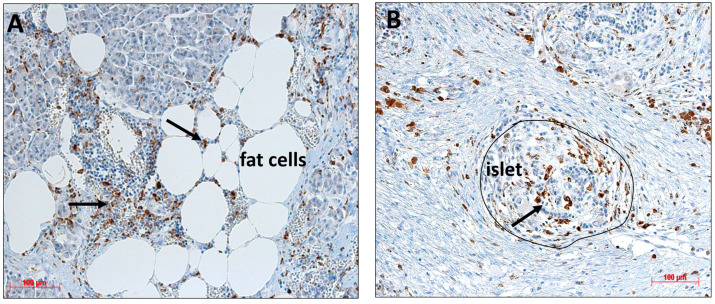
(**A**,**B**) Immunohistological staining of monocytes/macrophages (brown, black arrow) in a pancreatic resection using an antibody against CD68. Immune cells are found (**A**) in the proximity of fat cells and islets, as well as occasionally (**B**) inside islets.

In our previous studies, we observed monocyte/macrophage infiltration in both the exocrine pancreas and islets, especially in the areas characterized by increased adipocyte infiltration [2]. Immunostaining with the monocyte/macrophage marker CD68 identified many macrophages distributed all over the pancreatic tissue, near fat cells (Figure 8A), close to islets, and inside islets (Figure 8B). In some areas, an accumulation of macrophages was visible (Figure 8A, thick arrow).

### 2.7. Effect of Fetuin-A on Monocyte Migration towards PPAs

**Figure 9:**
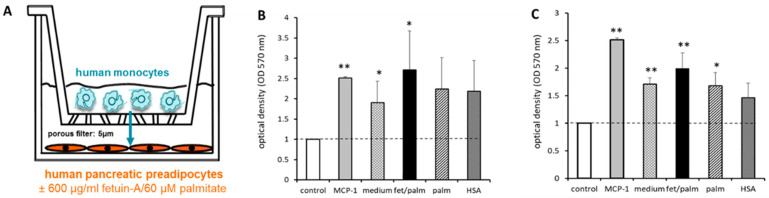
(**A**) Migration assay: human monocytes seeded on the upper filter side of a Transwell system were co-cultured with human PPAs-NGT or PPAs-T2D on the bottom of the plate. Then, 600 µg/mL fetuin-A ± 60 µmol/L palmitate were added to the culture medium of the lower compartment. MCP-1 was used as a ‘positive’ control (grey bars). (**B**) Results for PPAs-NGR. (**C**) Results for PPAs-T2D. All results are compared to untreated PPAs without monocytes (controls) cultured with standard culture medium (white bars). The third column shows the co-culture of PPAs with monocytes in standard culture medium and the fourth column after treatment with fetuin-A/palmitate. Data are expressed as the mean ± SEM (*n* = 3, Student’s *t*-test, * *p* < 0.05, ** *p* < 0.01).

Since PPAs and PAs might promote immune cell infiltration, we hypothesized that fetuin-A/palmitate may increase monocyte infiltration into the pancreas via stimulation of chemokine production, i.e., MCP-1, of the pancreatic fat cells. Migrating immune cells can induce matrix degradation and are involved in ECM remodeling [2,14]. In the Transwell system (Figure 9A), the potent chemoattractant MCP-1 strongly stimulated monocyte migration. A significant increase of monocyte migration was already observed in the presence of fat cells and human serum albumin. After the addition of fetuin-A/palmitate, monocyte migration was further stimulated. When PPAs-NGR (Figure 9B) were compared to PPAs-T2D (Figure 9C) in the migration assay, the stimulatory effect of fetuin-A/palmitate on monocyte migration was statistically more pronounced in PPAs-T2D (Figure 9C).

## 3. Discussion

In the present study, we investigated the expression of various ECM components in pancreatic resections, specifically in isolated human pancreatic fat cells and in human islets. We evaluated differences between PPAs isolated from subjects with NGR, PD, and T2D. Furthermore, the effects of the diabetogenic factors fetuin-A and palmitate on mRNA and protein expression of different ECM components. Since, in pancreatic resections, PAs are detected in close vicinity of islets, co-cultures were also performed in order to mimic the interplay between fat cells and islets. Since we detected immune cells in many pancreatic tissue resections, and monocytes/macrophages are known to produce ECM proteins and their degrading factors, we also examined monocyte migration under the influence of co-cultured fat cells and fetuin-A/palmitate administration.

We found that human pancreatic fat cells and islets express a large panel of different ECM components, degrading proteinases and inhibitors, as well as growth factors, which may influence cell viability, PPA differentiation, as well as hyperplasia and hypertrophy of fat cells. It is well known that fat cells are embedded in a unique ECM, which is not only responsible for mechanical support but participates in many signaling processes [21]. During adipose tissue expansion, remodeling of the ECM is necessary to enable adequate adipocyte growth [22,23]. Furthermore, constant adaptation to the fat cell volume is of major importance [24,25,26]. In obesity, adipose tissue alterations can occur due to inflammation and ECM turnover [24,25]. Adipose tissue expands either through hyperplasia or hypertrophy of existing adipocytes [27]. For hypertrophy, considerable tissue remodeling is required [28,29]. If the ECM does not permit adequate expansion, adipocytes are prone to necrosis [30] and cell death [31]. Remodeling of the ECM is characterized by proteolytic degradation involving matrix metalloproteinases (MMP), restructuring via ECM synthesis [24] and flexible adaptation of the ECM, which is enabled by increased elastin production [32]. Fat cells are also influenced by other cell types infiltrating the adipose tissue, e.g., macrophages and vascular cells [33,34,35], and by hormones, cytokines, fatty acids, and hepatokines that circulate systemically [2,6,36,37]. Currently, it is not clear how metabolic stress [38,39] impacts on ECM formation and degradation, and vice versa, in patients with fatty liver and/or fatty pancreas. Furthermore, the role of macrophages in this process is of major importance, serving as a critical source for ECM components and as modifiers of ECM during adipose tissue remodeling [26,35].

We investigated whether PPAs are ‘primed’ to express a different pattern or amount of ECM proteins depending on the presence or absence of hyperglycemia. In general, we observed no major differences in the basal mRNA expression levels of structural matrix components between subjects with different levels of glycemia. However, laminin (basal membrane) was more highly expressed in PPAs-T2D compared to PPAs-NGR. Furthermore, we found a lower mRNA level of the MMP inhibitor TIMP-2 in PPAs-T2D compared to PPAs-NGR. MMP imbalance is thought to be involved in the pathophysiology of obesity and T2D [24,40]. Increased plasma concentrations of MMP-2 and MMP-9 were found in patients with obesity and T2D [41,42]. 

The expansion of adipose tissue is associated with adipogenesis and angiogenesis, and it was shown that MMPs and their endogenous inhibitors are involved in these processes [43]. There is limited information about the role of TIMPs in the pathophysiology of T2D and obesity [44]. Most studies investigated their role in complications of T2D, such as cardiovascular diseases [45], foot ulcerations [46], or diabetic nephropathy [47]. A clinical study found that pro-MMP-9 levels were elevated, whereas TIMP-1 and TIMP-2 levels were reduced, in patients with T2D, compared to controls with NGR. Furthermore, MMP-3/TIMP-1 and the MMP-3/TMP-2 ratios were found to be higher in patients with T2D. In addition, fasting plasma glucose levels were found to be inversely correlated with TIMP-1 and TIMP-2 levels, but not to associate with MMP levels [48]. 

In the present study, we also found that the mRNA level of TGF-ß1 was higher in PPAs-T2D compared to PPAs-NGR. Increased serum TGFβ levels were detected in patients with obesity and T2D [49], and adipose expression of TGFβ was increased in obesity [50,51]. TGF-β1 promotes the proliferation of PPAs, ECM synthesis, and inhibits their differentiation into PAs [50]. It was also shown that PPAs exposed to TGF-β1 synthesize more ECM and are resistant to differentiation-inducing stimuli [49]. 

Our present data also indicate that the fatty liver, via fetuin-A, may influence the expression of a variety of ECM components in the fatty pancreas, in an interplay with palmitate. Fetuin-A/palmitate application to PPAs resulted in the downregulation of some ECM components. The most striking effects were found for fibronectin mRNA expression and protein secretion, which was reduced independently of the glycemic status of the subjects. Fibronectin impacts actin assembly and cell morphology and inhibits the differentiation of PPAs and lipogenesis [52]. 

We also found inhibitory effects of fetuin-A/palmitate on the proteoglycan decorin, which was shown to interact with several ECM components, including structural proteins, fibronectin and elastin, and growth factors such as TGF-β1. Besides stabilizing the extracellular matrix, these interactions may regulate the activity of growth factors and their receptors. Decorin may also influence metabolism and adipose tissue expansion through its function as a receptor on adipocyte progenitors for some adipokines, e.g., resistin [53].

Finally, fetuin-A/palmitate also exerted effects on the MMP inhibitors TIMP-1 and -2 in fat cells isolated from patients with PD or T2D. The regulation of MMP and TIMP expression is involved in adipocyte differentiation and the control of adipogenesis. Experimental studies showed that mice deficient in MMP-2 and membrane type-1 MMP have a lipodystrophic phenotype [54]. It can be speculated that the diabetogenic milieu in our study impairs the balance between matrix-degrading enzymes and their respective inhibitors, altering adipogenesis in vivo. In our in vitro study, the mRNA level of TGF-β1 was also downregulated by fetuin-A/palmitate in fat cells isolated from patients with PD and T2D. TGF-β1 is able to promote the proliferation of PPAs while inhibiting their differentiation into PAs and TGF-ß1 is known as an inhibitor of adipogenesis [55]. Thus, lower pancreatic TGF-ß1 levels may favor pancreatic adipogenesis in patients with PD or T2D. 

Analysis of histological sections of human pancreatic tissue revealed that fat cells are in close contact with islets [2,3]. An increased or decreased ECM production by fat cells may influence adjacent islets’ function. Matrix proteins, such as collagens, laminin, and fibronectin, can improve islet, and specifically beta cell, function [56]. The formation of matrix scaffolds is one of the important mechanisms enhancing islet survival. Col VI is a major component of the islet–exocrine interface, which is involved in islet-cell survival. We could detect some ECM proteins not just around but also inside islets. Pancreatic islets contain almost all major ECM components in varying amounts, predominantly laminins, fibronectin, and Col IV and VI. They are not only necessary as a structural support, but more importantly, are required for beta cell adhesion, proliferation, and insulin secretion [56]. In our previous study, we characterized pancreatic adipose tissue and examined the effect of pancreatic fat cells on islet function in a co-culture model. We found that fetuin-A/palmitate augments the inflammatory response of PPAs, which was further enhanced by the co-cultured islets [2,3]. 

Consequently, in the present study, we also investigated the expression of ECM components in human islets in mono- and co-cultures and a potential influence of fetuin-A/palmitate on this process. We found that several collagen types, as well as laminin, elastin, MMPs, TIMPs, and fibronectin, are also expressed in islets. We also detected the expression of the matrix components usherin and dermatopontin. Fetuin-A/palmitate treatment increased the mRNA expression of MMP-1, usherin and dermatopontin mRNA expression in co-cultured islets. There is limited information about the physiologic role of usherin in islets. However, in other tissues such as murine testicular basement membranes, an usherin/Col IV interaction was found to be required for the integrity of the basement membrane superstructure [57]. Moreover, dermatopontin, an extensively distributed non-collagenous component of the ECM, interacts with fibronectin, promotes fibronectin fibril formation, and enhances cell adhesion [58]. Dermatopontin was also found to modify the action of TGF-ß through interaction with decorin in the microenvironment of the ECM in vivo [59]. 

In a previous study, we found infiltration and accumulation of immune cells, predominately macrophages, next to fat cells and islets [2]. Moreover, we found that fetuin-A and palmitate induced chemokine (MCP-1 and IL-8) and cytokine (IL-6) production in human pancreatic fat cells, indicating that the fatty liver-derived fetuin-A is an important player in pancreatic fat cell inflammation [60]. Monocytes/macrophages are important sources of both cytokines and chemoattractants, such as MCP-1 [61]. The results of our transmigration assay indicate that the presence of fat cells (in co-culture) already stimulated monocyte migration. Fetuin-A/palmitate moderately enhanced this effect. Since MCP-1 and IL-8 mRNAs were highly expressed in pancreatic fat cells and their expression was further increased by fetuin-A/palmitate, we hypothesize that these two proteins are major chemoattractants for monocyte migration. Since immune cell infiltration requires matrix degradation [24,62], a higher amount of MMPs and a downregulation of structural ECM proteins and basal membrane components by fetuin-A/palmitate may favor the accumulation of immune cells in pancreatic fat tissue and in the islets [60]. Our findings are supported by a previous study in human PAs treated with macrophage-conditioned medium. A strong induction of MMP-1 and other MMPs was found, which suggests that macrophages stimulate tissue remodeling [63]. A limitation of our co-culture studies is the low statistical power of these experiments due to the low number of co-culture experiments that could be performed. This is based on the fact that it is very difficult to simultaneously obtain a sufficient number of human PPAs and human islets. 

## 4. Materials and Methods

### 4.1. Human Pancreatic Resections

Pancreatic resections from a tumor-free region of patients who underwent pancreatic surgery were used for this study. We studied pancreatic resections and isolated PPAs of patients with NGR, PD, or T2D based on the fasting plasma glucose levels and the HbA1c values (Table 2) using the respective cut-off values as recommended by the American Diabetes Association [64].

### 4.2. Immunohistological Stainings 

Pancreatic tissue samples were fixed in formalin, embedded in paraffin, and 4 μm-thick serial sections were performed. Haematoxylin-eosin (HE, Sigma, Taufkirchen, Germany), Elastica–van–Gieson (EVG, Sigma, Taufkirchen, Germany) and immunostainings with specific rabbit polyclonal antibodies against collagen IV and VI (OriGene EU, Herford, Germany) were conducted with automated protocols [2]. Primary antibodies were detected using the Opti-View Kit, which is a hapten-multimer system without biotin (Roche–Ventana, Basel, Switzerland). 

### 4.3. Isolation, Culture, Differentiation, and Treatment Conditions of Human PPAs and Pancreatic Islets

PPAs were isolated from adipose tissue obtained from the pancreatic resections, as previously described [2]. Patients undergoing pancreatic surgery provided written informed consent to donate pancreas tissue for research purposes. We obtained macroscopically healthy tissue that had been resected during surgery but was not needed for further pathology workup [2].

Isolated PPAs were expanded in MEM/Ham’s mixture F12 (1:1, Fisher Scientific GmbH, Schwerte, Germany) containing 20% FCS, 1% chicken embryo extract (Sera Laboratories, West Sussex, UK), 2 mmol/L glutamine, 100 IU/mL penicillin, 0.1 mg/mL streptomycin, and 0.5 mg/mL fungizone. PPAs differentiation to PAs was conducted for 7 d in DMEM/Ham’s mixture F12 (1:1, Fisher Scientific GmbH, Schwerte, Germany) containing 5% FCS (Biochrom, Berlin, Germany), 17 µmol/L pantothenate, 1 µmol/L biotin (Roth, Karlsruhe, Germany), 2 mmol/L glutamine, 2 µg/mL apotransferrin, 1 µmol/L insulin (Sanofi-Aventis Deutschland GmbH, Frankfurt, Germany), 100 IU/mL penicillin, 0.1 mg/mL streptomycin, and 0.5 mg/mL fungizone and supplemented with 0.5 mmol/L 3-isobutyl-1-methyl-xanthine, 1 µmol/L cortisol, 5 µmol/L troglitazone, and 50 µmol/L indomethacin (Biochrom, Berlin, Germany and Sigma Taufkirchen, Germany). The cells were terminally differentiated, as previously described [65]. Human islets were procured through the European Consortium for Islet Transplantation (ECIT) and were cultured in CMRL-1066 containing, in mmol/L, 5 glucose, 2 L-glutamine, and 10 HEPES and supplemented with 10% FCS (Biochrom, Berlin, Germany), as previously published [2]. Treatment with 0.6 mg/mL human fetuin-A (Sigma-Aldrich, Munich, Germany), 0.6 mg/mL human serum albumin (hSA, CLS Behring GmbH, Marburg, Germany), as control, and 60 μmol/L palmitate (Sigma-Aldrich, Munich, Germany) was performed in FCS-free culture medium (in order to avoid exposure to bovine fetuin-A). The concentrations of palmitate were adapted to the concentration of hSA. Fetuin-A concentration of 0.6 mg/mL matches plasma levels in hyperglycemic humans [66,67].

### 4.4. Co-Culture of Human Pancreatic Fat Cells and Islets

Co-culture experiments were performed as previously described [2]. Half of the mono- and co-cultures were treated with fetuin-A (alpha-2-HS-glycoprotein, Sigma, München, Germany, endotoxin <0.05 EU/μg [0.005 ng/μg] determined by ELISA kit Hycult Biotech, Uden, the Netherlands). PPAs-PD were subcultured in 6-well plates, and the human islets were placed in inserts of a Transwell system, which allows for the separate quantification of mRNA expression in fat cells and islets, as well as determination of protein levels in the media from the two distinct compartments [2]. Mono- and co-cultures were performed for 24 h in FCS-free human islet culture medium supplemented with fetuin-A, human serum albumin (hSA) and palmitate.

### 4.5. Gene Expression

The cellular RNA of fat cells and islets was isolated (Nucleo Spin RNAII, MACHEREY-NAGEL GmbH & Co. KG, Düren, Germany) and transcribed using random primers (Eurofins Genomics, Ebersberg, Germany, Supplementary Table S1). Measurements were performed with the Light Cycler 480 System (Roche, Mannheim, Germany). Normalized gene expression was calculated as a ratio of cycle threshold values (Ct) of target vs. housekeeping gene (Rps13) transcripts (2^−ΔCt^), as previously described [2]. 

### 4.6. Transcriptome Analysis (RNAseq)

Transcriptome analysis was performed as previously described [3,65]. Briefly, aliquots of preadipocytes and adipocytes were used for RNA extraction, and mRNA was isolated from 1 µg RNA by poly-dT enrichment using the NEBNext Poly(A) mRNA Magnetic Isolation Module (BioLabs, Heidelberg, Germany). After fragmentation, the samples were subjected to the workflow for strand-specific RNAseq library preparation (Ultra Directional RNA Library Prep II, NEB) and 75 bp single-read sequencing was performed on an Illumina NextSeq500 (Shirley, NY, USA). After sequencing, FastQC was used to perform quality control. Differential expression between preadipocytes and adipocytes was tested with the R package DESeq2 v2.7.R [65,68]. Selected gene expression data have been published previously [3,65]. The complete data set is available under GEO accession GSE169514. A fold-change of ≥2 and ≤−2 and Benjamini–Hoch-berg-adjusted *p* values of ≤0.05 were used as criteria to select differentially expressed genes (DEG).

### 4.7. Quantification of Protein Secretion

Luminex xMAP^®^ technology (Luminex Corporation, Austin, TX, USA) was used to quantify protein concentrations in supernatants. A customized Human Magnetic Luminex Assay (R&D systems 9-Plex, Bio-Techne GmbH, Wiesbaden-Nordenstadt, Germany) with the following analytes was used: ADAMTS13 (BR52) Collagen I alpha 1 (BR18) Collagen IV alpha 1 (BR55) Fetuin A/AHSG (BR57) Fibronectin (BR56). Furthermore, MMP-1, MMP-2, MMP-10, TIMP-1, TIMP-2, and TIMP-3 protein levels were determined by Milliplex (Merck KGaA Darmstadt, Germany): MAP Human MMP Panel 2 Magnetic (No. HMMP2MAG-5) and MAP Human TIMP Panel 1 Magnetic (No. HTMP1MAG-54K).

### 4.8. Monocyte Migration Assay

Monocytes were isolated through the positive selection of CD14+ cells directly from anticoagulated whole blood [69] using the autoMACS Pro Separator, the MultiMACS Cell24 Separator Plus (Miltenyi Biotec B.V. & Co. KG, Bergisch Gladbach, Germany), or the Whole Blood Column Kit (Whole Blood CD14 MicroBeads human Order no. 130-090-879). Isolated monocytes were seeded on top of a filter insert hanging in a Transwell chamber above PPAs monocytes, and PPAs were co-cultured for 24 h [69]. Transmigration of monocytes was quantified by the Chemotaxis Cell Migration Assay (QCM Chemotaxis Cell Migration Assay, 24-well, 5 µm, Merck KGaA Darmstadt, Germany). After chemotaxis, migration of monocytes on the lower filter side was measured colorimetrically (560 nm) following staining with crystal violet, a nucleic dye. The mRNA levels of MCP-1 and IL-8 were determined in co-cultured PPAs by real-time PCR, as previously described [2]. 

### 4.9. Study Approval

All experiments with human material were approved by the Ethics Committee of the Medical Faculty and the University Hospital of the University of Tübingen, Germany (No. 697/2011BO1 and 355/2012BO2). All patients provided informed written consent. 

### 4.10. Statistics

Data are expressed as the mean ± SD or SEM. Statistical analysis was performed using GraphPad Prism 9.1.2 (226) (GraphPad, La Jolla, CA, USA). The differences between groups were determined by unpaired, 2-tailed Student’s *t*-test or ANOVA with Holm-Šídák method post-hoc test correction as appropriate. An adjusted * *p* < 0.05 was considered statistically significant. Detailed *p*-values are indicated in the respective figure legends.

## 5. Conclusions

We provide novel information that several ECM components are expressed in human pancreatic fat cells and islets and that the mRNA levels of distinct ECM components differ in PPAs-PD and PPAs-T2D compared to PPAs-NGR. Furthermore, our data indicate that fetuin-A/palmitate alters the balance between ECM formation and breakdown, which may result in remodeling of the matrix structure of the pancreatic tissue and islets. Finally, the accumulation of immune cells may trigger these processes and stimulate pancreatic inflammation. 

## Figures and Tables

**Table 1:** Summary of the results of the mRNA expression of ECM components and their regulators in preadipocytes and in adipocytes, respectively. The metabolic status of the patients is indicated by NGR = normal glucose regulation, PD = prediabetes, D = type 2 diabetes, SP = structural proteins, BM = components of the basement membrane, GF = growth factors, and EZ = enzymes (metalloproteinases and inhibitors). The expression data for Col V, tenascin, elastin, and MMP-10 were very low or below detection limit and, therefore, were not considered. n.s. indicates non-significance; one arrow indicates * *p* < 0.05 and two arrows indicate ** *p* < 0.01, The number of arrows correspond with the number of * or **.

		Metabolic Status			PA vs. PPA			Fetuin/Palm			
		PD vs. NGR	D vs. NGR	D vs. NGR	NGR	PD	D	NGR	PD	D	Pooled
SP	Col I	n.s.	n.s.	n.s.	n.s.	n.s.	n.s.	n.s.	n.s.	n.s.	n.s.
	Col III	n.s.	n.s.	n.s.	n.s.	n.s.	n.s.	n.s.	n.s.	n.s.	n.s.
	Col VI	n.s.	n.s.	n.s.	n.s.	n.s.	n.s.	n.s.	n.s.	n.s.	n.s.
	FN	n.s.	n.s.	n.s.	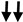	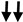	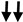	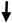	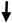	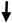	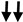
	Decorin	n.s.	n.s.	n.s.	n.s.	n.s.	n.s.	n.s.	n.s.	n.s.	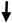
BM	Col IV	n.s.	n.s.	n.s.	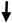	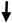	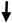	n.s.	n.s.	n.s.	n.s.
	LN	n.s.	n.s.	n.s.	n.s.	n.s.	n.s.	n.s.	n.s.	n.s.	n.s.
GF	TGF-β1	n.s.	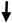	n.s.	n.s.	n.s.	n.s.	n.s.	n.s.	n.s.	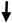
	CTGF	n.s	n.s.	n.s.	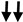	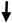	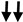	n.s.	n.s.	n.s.	n.s.
EZ	MMP-1	n.s.	n.s.	n.s.	n.s.	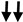	n.s.	n.s.	n.s.	n.s.	n.s.
	MMP-2	n.s.	n.s.	n.s.	n.s.	n.s.	n.s.	n.s.	n.s.	n.s.	n.s.
	MT-MMP	n.s.	n.s.	n.s.	n.s.	n.s.	n.s.	n.s.	n.s.	n.s.	n.s.
	TIMP-1	n.s.	n.s.	n.s.	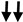	n.s.	n.s.	n.s.	n.s.	n.s.	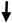
	TIMP-2	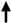	n.s.	n.s.	n.s.	n.s.	n.s.	n.s.	n.s.	n.s.	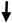
	TIMP-3	n.s.	n.s.	n.s.	n.s.	n.s.	n.s.	n.s.	n.s.	n.s.	n.s.

**Table 2:** Anthropometric and clinical data of human pancreatic fat donors. Results expressed as the median (SD). ^1^ Available from 8 subjects; ^2^ Available from 6 subjects. Groups that are not connected by the same letter differ significantly (*p* < 0.05); Student’s *t*-test. P^1^ *p*-value; one-way ANOVA. NGR: normal glucose regulation; PD: prediabetes; T2D: type 2 diabetes.

Trait	NGR	PD	T2D	P^1^
N (% males)	8 (88)	7 (57)	9 (67)	0.444
Age (years)	61 (12.56)	65 (12.13)	66 (12.85)	0.454
BMI (kg/m^2^)	26.57 (3.57)	25.54 (2.61)	27.03 (5.77)	0.792
Fasting glucose (mmol/L)	5.13 (0.71) ^a,1^	4.81 (0.79) ^a^	7.67 (2.30) ^b^	0.003
HbA1c (%)	5.46 (0.17) ^a,1^	5.84 (0.10) ^a^	7.19 (1.00) ^b^	0.0001
Fasting insulin (pmol/L)	66.29 (40.96) ^1^	54.14 (27.93)	62.63 (30.06) ^2^	0.784

## Data Availability

Not applicable.

## Supplementary Materials

The supporting information can be downloaded at: https://www.mdpi.com/article/10.3390/ijms241311169/s1.
