# Supplementary material for: Extracellular Matrix Expression in Human Pancreatic Fat Cells of Patients with Normal Glucose Regulation, Prediabetes and Type 2 Diabetes
Source: Int J Mol Sci. 2023 Jul 6;24(13):11169. doi: 10.3390/ijms241311169 (PMC10342602; doi:10.3390/ijms241311169)

Figure S1: Relative mRNA levels in PPAs after treatment with 600 µg/ml fetuin-A + 60 µmol/L palmitate for 24h (black bars) versus untreated controls (white bars). **(A)** In PPAs isolated from subjects with NGR, **(B)** in PPAs isolated from subjects with PD and **(C)** in PPAs isolated from subjects with T2D. ΔCT values are shown (related to the housekeeping gene *RPS13*. Data are expressed as mean ± SEM (n=5-7, student's *t*-test, *$p<0.05$).

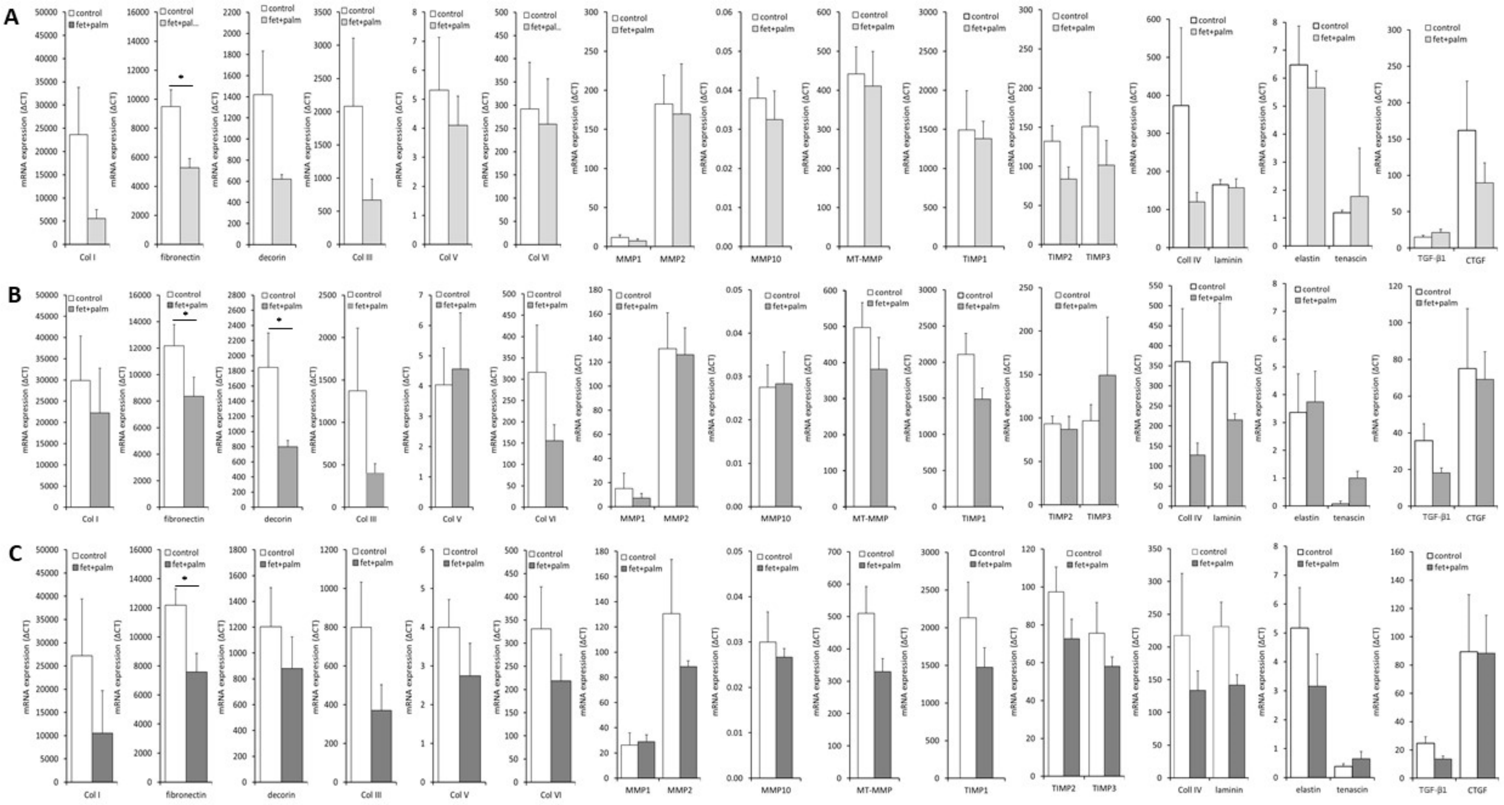

Supplement: Supplementary file 1 [file ijms-24-11169-s001.zip › Supplementary Figure S1.pdf]
