# Supplementary material for: Extracellular Matrix Expression in Human Pancreatic Fat Cells of Patients with Normal Glucose Regulation, Prediabetes and Type 2 Diabetes
Source: Int J Mol Sci. 2023 Jul 6;24(13):11169. doi: 10.3390/ijms241311169 (PMC10342602; doi:10.3390/ijms241311169)
Supplement: Supplementary file 1 [file ijms-24-11169-s001.zip › Supplementary Table S1.pdf]

Gene	Forward primer sequence	Reverse primer sequence	Probe
**COL I**	5-TATTGCTGGACAGCGTGGT-3	5-ACCTTGTTTGCCAGGTTCAC-3	72
**COL III**	5-GAGGCCCTCCTGGAGTTG-3	5-GGAAGACCACGAGCACCA-3	62
**COL VI**	5-GAAGAGAAGGCCCCGTTG-3	5-CGGTAGCCTTTAGGTCCGATA-3	80
**USH2A**	5-GACACAACAAGCCAGCCATA-3	5-AGAGGCAAGCAGCGATCA-3	83
**DPT**	5-CGAGGAGCAACAACCACTTT-3	5-CGGCACATTATGAACTTCCA-3	65
**FN**	5-ACTGGAGTGCTCACAGTCTCC-3	5-CGTTTGTAGGGGTTGTGGTAAT-3	55
**Decorin**	5-CCAATATCACCAGCATTCCTC-3	5-TTGTTGCCATCAAGATGTAATTC-3	69
**COL IV**	5-GACCCCCGGGAGAAATAG-3	5-TTTGAAAAAGCAATGGCACTC-3	37
**Laminin**	5-TGTCAAGAGCATGGATAATGAAA-3	5-CTAGGGGAAGGTGTGCTCTG-3	65
**TGF-ß1**	5-GCAGCACGTGGAGCTGTA-3	5-CAGCCGGTTGCTGAGGTA-3	72
**cTGF**	5-AAAATTTTAGCGTGCTCACTGAC-3	5-TCAGTCTCTTGATGGCTGGA-3	45
**MMP-1**	5-GCATATCGATGCTGCTCTTTC-3	5-GATAACCTGGATCCATAGATCGTT-3	47
**MMP-2**	5-CCCCAAAACGGACAAAGAG-3	5-CTTCAGCACAAACAGGTTGC-3	43
**MT-MMP**	5-CAGGAATGAGGATCTGAATGG-3	5-CCGAGGGGTCACTGGAAT-3	45
**RPS13**	5-CCCCACTTGGTTGAAGTTGA	5-ACACCATGTGAATCTCTCAGGA-3	68
**TIMP-1**	5-GGCTGTGAGGAATGCACA-3	5-TGGAAGCCCTTTTCAGAGC-3	17
**TIMP-2**	5-GAAGAGCCTGAACCACAGGT-3	5-CGGGGAGGAGATGTAGCAC-3	43
**TIMP-3**	5-GCTGGAGGTCAACAAGTACCA-3	5-CACAGCCCCGTGTACATCT-3	62

Supplementary Table S1: Homo sapiens primers and probes for PCR amplification
